# How does the age of control individuals hinder the identification of target genes for Huntington’s disease?

**DOI:** 10.3389/fgene.2024.1377237

**Published:** 2024-06-20

**Authors:** João Rafael Dias Pinto, Benedito Faustinoni Neto, Joyce Macedo Sanches Fernandes, Irina Kerkis, Rodrigo Pinheiro Araldi

**Affiliations:** ^1^ BioDecision Analytics Ltda., São Paulo, Brazil; ^2^ Post-Graduation Program in Structural and Functional Biology, Paulista School of Medicine (EPM), Federal University of São Paulo (UNIFESP), São Paulo, Brazil; ^3^ Medical Physiology Department, State University of Campinas (UNICAMP), Campinas, Brazil; ^4^ Genetics Laboratory, Instituto Butantan, São Paulo, Brazil

**Keywords:** RNA-seq analysis, Huntington’s disease, case-control, aging, PSM, bioinformatics

## Abstract

Several studies have compared the transcriptome across various brain regions in Huntington’s disease (HD) gene-positive and neurologically normal individuals to identify potential differentially expressed genes (DEGs) that could be pharmaceutical or prognostic targets for HD. Despite adhering to technical recommendations for optimal RNA-Seq analysis, none of the genes identified as upregulated in these studies have yet demonstrated success as prognostic or therapeutic targets for HD. Earlier studies included samples from neurologically normal individuals older than the HD gene-positive group. Considering the gradual transcriptional changes induced by aging in the brain, we posited that utilizing samples from older controls could result in the misidentification of DEGs. To validate our hypothesis, we reanalyzed 146 samples from this study, accessible on the SRA database, and employed Propensity Score Matching (PSM) to create a “virtual” control group with a statistically comparable age distribution to the HD gene-positive group. Our study underscores the adverse impact of using neurologically normal individuals over 75 as controls in gene differential expression analysis, resulting in false positives and negatives. We conclusively demonstrate that using such old controls leads to the misidentification of DEGs, detrimentally affecting the discovery of potential pharmaceutical and prognostic markers. This underscores the pivotal role of considering the age of control samples in RNA-Seq analysis and emphasizes its inclusion in evaluating best practices for such investigations. Although our primary focus is HD, our findings suggest that judiciously selecting age-appropriate control samples can significantly improve best practices in differential expression analysis.

## 1 Introduction

Huntington’s disease (HD) is a fatal autosomal dominant neurodegenerative disorder characterized by an expanded trinucleotide CAG (cytosine-adenine-guanine) repeat in exon 1 of the *HTT* (huntingtin) or IT15 gene (located at 4p.16.3) (MacDonald, 1993; Kerkis et al., 2022; Jiang et al., 2023). The length of the CAG expansion significantly influences the age of onset. Typically falling within the range of 40–50 CAG repeats, the onset age varies from 30 to 65 years for most affected individuals (MacDonald, 1993; Kerkis et al., 2022; Jiang et al., 2023). CAG lengths beyond this range are frequently associated with juvenile-onset, while CAG lengths of 36–39 are linked to partial penetrance and delayed onset of the disease (MacDonald, 1993; Kerkis et al., 2022).

A high number of CAG repeats (>36) encodes a mutated huntingtin (mHTT) protein, which possesses an expanded polyglutamine (polyQ) tract. The dysfunctional mHTT protein can forms aggregates within neurons and other cells (DiFiglia et al., 1995; 1997; Lee et al., 2019; Ferguson et al., 2022; Castro et al., 2023), and leads to (i) impairment of the ubiquitin-proteasome pathway (reducing mHTT detoxification), (ii) transcriptional dysregulation, (iii) excitotoxicity (due to increased glutamate and glutamate agonist release from cortical afferents), (iv) mitochondrial dysfunction and altered energy metabolism, and (v) changes in axonal transport and synaptic dysfunction (Araldi et al., 2022; Castro et al., 2023). Collectively, these dysregulations contribute to motor, neuropsychiatric, and cognitive impairments observed in HD patients (Hong et al., 2021; Kerkis et al., 2022; Wenceslau et al., 2022).

While the pathophysiological mechanisms of the disease are well understood, there is currently no approved treatment capable of delaying or preventing the progressive neuronal death caused by HD (Barker and Mason, 2019; Kerkis et al., 2022). The absence of disease-modifying therapy for HD patients can be attributed to the absence of (i) prominent pharmaceutical targets for drug development, (ii) suitable animal models to assess the potential therapeutic benefits of investigational products, and (iii) prognostic biomarkers to demonstrate the potential advantages of investigational products in clinical trials.

In the pursuit of identifying pharmaceutical targets and/or prognostic biomarkers, numerous studies have already sequenced the transcriptome of brain regions implicated in HD pathophysiology (Labadorf et al., 2015; Labadorf et al., 2016; Lin et al., 2016; Agus et al., 2019). Specifically, these investigations have concentrated on Brodmann Area 9 (BA9, dorsolateral pre-frontal cortex) or BA4 (primary motor cortex), as well as the caudate nucleus (CAU), as illustrated in Table 1. This is because about 90% of striatal neurons, primarily affected by the disease, are lost in late-stage disease (DiFiglia et al., 1997; Aylward et al., 2004; Aylward et al., 2011). This makes it difficult to study striatal postmortem samples from individuals with HD due to the scarcity of neurons in this highly degenerated tissue (Labadorf et al., 2015; Agus et al., 2019). However, studies based on structural magnetic resonance imaging (MRI) evidenced that, in late-stage HD, BA9 exhibits loss of projection neurons in layers III, V, and VI and glial density increase in deeper layer (VI) consistent with cortical degeneration (Selemon et al., 2004; Delmaire et al., 2013). These results make the BA9 an important brain area to be explored to identify possible pharmacological/prognostic targets for HD.

**TABLE 1:** BioProjects available on SRA public database which analyzed the transcriptome of brain areas from Huntington’s disease affected individuals.

		Number of samples				
BioProject	Area	Total	Control	HD	Data volume (Tb)	Reference
PRJNA271929	BA9	69	49	20	0.38	Labadorf et al. (2015)
						Labadorf et al. (2016)
PRJNA670925	BA9	100	68	32	0.26	Labadrof et al. (2018)
PRJNA531456	BA9 CAU	85	54	31	0.46	Agus et al. (2019)
PRJNA316625	BA4	14	7	7	0.25	Lin et al. (2016)
Total samples		268	110	158	1.35	

With the advances in bioinformatics, these samples have undergone extensive reanalysis through various pipelines (Seefelder and Kochanek, 2021; Sneha et al., 2023). Despite the commendable efforts invested in these studies, the differentially expressed genes (DEGs) identified so far have not yielded valuable pharmaceutical or prognostic targets conducive to HD drug development (Labadorf et al., 2015; 2016; Lin et al., 2016; Agus et al., 2019; Seefelder and Kochanek, 2021; Sneha et al., 2023).

Upon analyzing these studies, it was observed that they compared the transcriptome of BA9/BA4 regions in individuals positive for the HD gene with that of older neurologically normal control individuals (matched in terms of mean age) (Labadorf et al., 2015; Labadorf et al., 2016; Lin et al., 2016; Agus et al., 2019). Nevertheless, it is well-established that aging induces notable transcriptome alterations in the brain, leading to changes in energy metabolism (Błaszczyk, 2020; Palmer and Jensen, 2022), diminished synaptic function (Fan et al., 2018; Temido-Ferreira et al., 2019), disruptions in the immune system with subsequent triggering of neuroinflammation (Finger et al., 2022; Andronie-Cioara et al., 2023), and accumulation of iron (Hagemeier et al., 2012; González-Velasco et al., 2020; Ham and Lee, 2020). These age-related factors collectively contribute to the exacerbation of neurodegenerative processes (Bowirrat, 2022). Consequently, it is no surprise that aging contributes to the gradual deterioration of physiological and biochemical functions, encompassing motor and cognitive decline (Ham and Lee, 2020). This phenomenon is notably observed in neurodegenerative disorders like HD (Domínguez et al., 2016; Barron et al., 2021; Jia et al., 2022; van de Zande et al., 2023; Wilton et al., 2023).

This observation prompted us to hypothesize that the inappropriate use of control samples from older, neurologically normal individuals (compared to HD gene-positive individuals) may lead to the misidentification of DEGs. We conducted a comparative reanalysis of transcriptomic data from BA9 tissue samples collected from 20 HD gene-positive individuals and 49 neurologically normal control individuals to test our hypothesis. The dataset utilized in this study was sourced from the Sequence Read Archive (SRA), a public database.

In assessing whether the utilization of older controls might result in DEG misidentification, we identified DEGs in HD gene-positive individuals concerning the entire control group (*n* = 49) and a “virtual” group of 20 neurologically normal individuals selected from the 49 control samples using propensity score matching (PSM). PSM is a non-parametric statistical technique employed to construct a control group by matching each affected unit with a non-affected unit of similar characteristics (Walsh et al., 2012; Kane et al., 2020).

Our results revealed that the use of older controls indeed leads to the misidentification of DEGs, negatively impacting the discovery of pharmaceutical and/or prognostic markers. This finding underscores the importance of considering the age of control samples in RNA-Seq analysis, suggesting that it should be included assessing of best practices for such investigations.

## 2 Material and methods

### 2.1 Ethical approval

This study utilized publicly accessible sequencing data obtained from HD gene-positive and neurologically normal individuals in the Sequence Read Archive (SRA) public repository database. Consequently, formal ethical approval was deemed unnecessary for this investigation.

### 2.2 Dataset description

The sequencing data of the dorsolateral pre-frontal cortex (BA9) from both HD gene-positive and control samples was obtained from the SRA database (BioProject PRJNA271929, available on https://www.ncbi.nlm.nih.gov/bioproject/PRJNA271929). This BioProject is comprised of 69 postmortem samples, featuring 20 from HD gene-positive individuals aged between 40 and 75 years (with a mean of 58.2 ± 10.4 years) and 49 from neurologically normal control human individuals aged between 36 and 106 years (with a mean of 68.3 ± 15.8 years). The selection of this BioProject for our study was based on the following criteria: (i) the presence of a satisfactory number of case-control samples, (ii) the high quality of sequencing data, and (iii) the prior analysis of these samples by Labadorf et al. (Labadorf et al., 2015), followed by subsequent reanalysis, including additional BA9 and/or CAU samples (Labadorf et al., 2018; Agus et al., 2019). Demographic data from the samples are described in Supplementary Excel S1.

### 2.3 Control sample selection by age distribution using the propensity score matching

To assess our hypothesis that the inappropriate use of older control samples can affect DEGs identification, we employed propensity score matching (PSM) at a 1:1 ratio (HD gene-positive subject: neurologically normal subject). PSM is a quasi-experimental method, initially introduced by Rosenbaum and Rubin (1983), that aims to align affected/treated and control groups based on a targeted feature to enhance comparability. For this purpose, we utilized the MatchIt package in R (Ho et al., 2007). To validate the outcomes derived from the package, the age distribution was visually examined before and after PSM through both boxplot and density plot analyses. Additionally, the statistical confirmation of age distribution equality was carried out using a t-Student test, all executed within the R environment.

### 2.4 Pre-processing (quality control and mRNA abundance estimation)

The RNA-Seq data, formatted in FASTQ, underwent pre-processing through FastQC (Andrews, 2010) and MultiQC (Ewels et al., 2016) tools to ensure sequencing quality. Subsequently, the pre-processed reads were mapped to the latest human genome (hg38) using STAR (Spliced Transcript Alignment to a Reference), a splice-aware aligner designed for accurately aligning reads to the reference genome (Dobin et al., 2013). Transcript abundance was then estimated utilizing the feature count read summarization program (Liao et al., 2014). The abundance estimates from all samples were consolidated into a unified expression matrix and normalized using the DESeq2 package v1.10.1 (Love et al., 2014), following the methodology outlined by Labadorf et al. (2015).

### 2.5 Differential gene expression analysis

To gain deeper insights into how the age of control samples may impact the identification of differentially expressed genes, the normalized read counts of HD gene-positive individuals (*n* = 20) were individually compared with both the entire control sample set (*n* = 49) and the PSM-selected control samples (*n* = 20, hereafter referred to as Age-matched control samples). The differential expression analysis was performed using the DESeq2 method (Love et al., 2014), generating a comprehensive list of genes along with their respective levels of differentiation and statistical significance.

**FIGURE 1:**
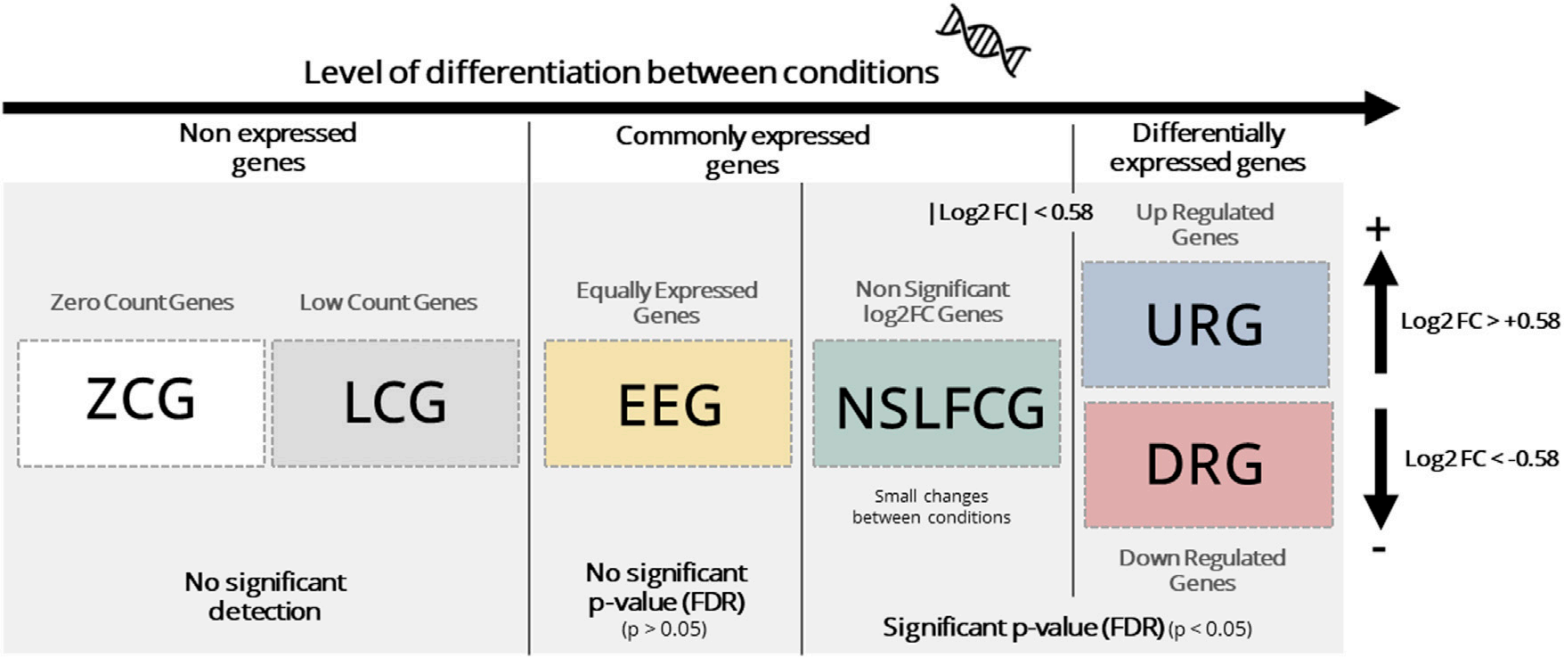
Gene classification. Genes are generically classified as: i) non expressed, which includes zero count genes (ZCG, row counts = 0) and low count genes (LCG, normalized counts < 10 in less than 70% of the smaller category), ii) commonly expressed, which includes equally expressed genes (EEG, adjusted *p*-value > 0.05) and non-significant log2FC (LFC) genes (NSLFCG, adjusted *p*-value < 0.05, but |log2FC| < 0.58) and, iii) differentially expressed genes (DEGs), which include upregulated genes (URG, adjusted *p*-value < 0.05 and log2FC > 0.58) and downer genes (DRG, adjusted *p*-value < 0.05 and log2FC < −0.58).

Mapped genes were categorized based on their abundance, *p*-value, and fold change (|log2FC|) into six distinct categories, as outlined in Table 2; Figure 1. Genes classified as upregulated (URG) or downregulated (DRG) were considered differentially expressed genes (DEGs), while other categories were designated as non-differentially expressed genes (NDEGs). Notably that both zero count genes (ZCG; genes with row counts = 0) and low count genes (LCG; genes with normalized counts < 10 in less than 70% of the smaller category) are excluded from the differential gene expression analysis using DESeq2.

**TABLE 2:** Gene classification according to differential expression in relation to the patients with Huntington’s disease.

Classification	Abbreviation	Reads	*p*-adjust	log2FC
Zero count gene^a^	ZCG	= 0	NA	NA
Low count gene^b^	LCG	<10	NA	NA
Equally expressed gene	EEG	≥10	>0,05	NA
Non**-**significant log2FC gene	NSLFCG	≥10	<0,05	| log2FC | < 0.58^c^
Upregulated genes	URG	≥10	<0,05	log2FC > 0.58
Downregulated genes	DRG	≥10	<0,05	log2FC < −0.58

^a^ Genes that showed reads equal zero across all samples (not identified).

^b^ Genes with reads >10 that accounts for less than 70% of a limit number of samples (here we adopt the number samples of the smallest category)); NA, not applicable.

^c^ | log2FC | > 0.58 or - 0.58 < log2FC < + 0.58 (which corresponds roughly to change less than ± 50% of the reference level).

### 2.6 Unsupervised dimension reduction and clustering analyses

For a comprehensive comparative assessment of the entire transcriptome between HD gene-positive individuals and all neurologically normal subjects, we employed the Uniform Manifold Approximation and Projection (UMAP) dimensionality reduction technique (McInnes et al., 2018), which proved itself to produce insightful reduced dimensions to represent genomic data (Dorrity et al., 2020). Utilizing three distinct components from UMAP, we subsequently applied the Density-based Spatial Clustering of Applications with Noise (DBSCAN) algorithm to unveil primary grouping patterns (Kriegel et al., 2011); the advantage of this approach is the fact that no prior knowledge on the ideal number of clusters is required; also, DBSCAN generates clusters based on density of data in a particular region and thus automatically recognizes anomalous or outlying observations. The UMAP analysis was performed in CRAN-R environment through UMAP package, while the subsequent application of DBSCAN was applied using the scikit-learn-based DBSCAN implementation for Python 2.6.

### 2.7 Functional enrichment analyses

Recognizing that the biological effects on the HD phenotype hinge on the intricate interplay among DEGs, we subjected the ranked list of genes—comprising both URGs and DRGs—derived from the analysis using all control samples (*n* = 49) and age-matched controls (*n* = 20) to distinct functional enrichment analyses. These analyses were conducted using the Gene Set Enrichment Analysis (GSEA) to explore biological pathways (BP) and molecular functions (MF).

### 2.8 Variation of genes abundance across age in the control samples

To assess the influence of aging on genes that have become DEGs or NDEGs, we employed a linear regression model on the median of the reads by age across all control samples. This approach allowed us to examine trends and determine their statistical significance. These analyses were conducted within the R environment, utilizing the stats package.

### 2.9 Analysis of the older neurologically normal individual's removal on the adjusted *p-*value

Given that the raw *p*-values obtained through the Wald test (employed for DEG identification by DESeq2) are subjected to correction for multiple testing using the Benjamin and Hochberg (BH) method to control the false-discovery rate (FDR) (Benjamin and Hochberg, 1995) and considering that age matching primarily affects the genes identified as DEGs, we further hypothesized that age matching could potentially influence DEG discovery by altering the p-adjusted values. To investigate this hypothesis, we compared the raw *p*-values and the p-adjusted values of genes that ceased to be identified as DEGs upon excluding control samples from older neurologically normal individuals.

## 3 Results

### 3.1 All samples show a satisfactory quality, making them eligible for downstream analyses

Quality control analysis stands as an indispensable facet of RNA-Seq analysis (Consortium, 2011; Conesa et al., 2016; Chung et al., 2021; Faustinoni-Neto et al., 2023). Therefore, we meticulously evaluated the quality control of the 69 BioSamples by utilizing FastQC and MultiQC tools. The outcomes unequivocally affirmed that all BioSamples within the BioProject PRJNA271929 exhibited a median per-sequence Phred score of 36, signifying a sequencing accuracy surpassing 99.9%. Furthermore, the samples displayed a %GC content of 49%, indicating the absence of potential contaminations. These findings reassert the high quality of all 69 BioSamples, aligning with prior validations conducted by Labadorf et al. (2015).

### 3.2 PMS appropriately selected control samples with age distribution similar to HD gene-positive individuals

**FIGURE 2:**
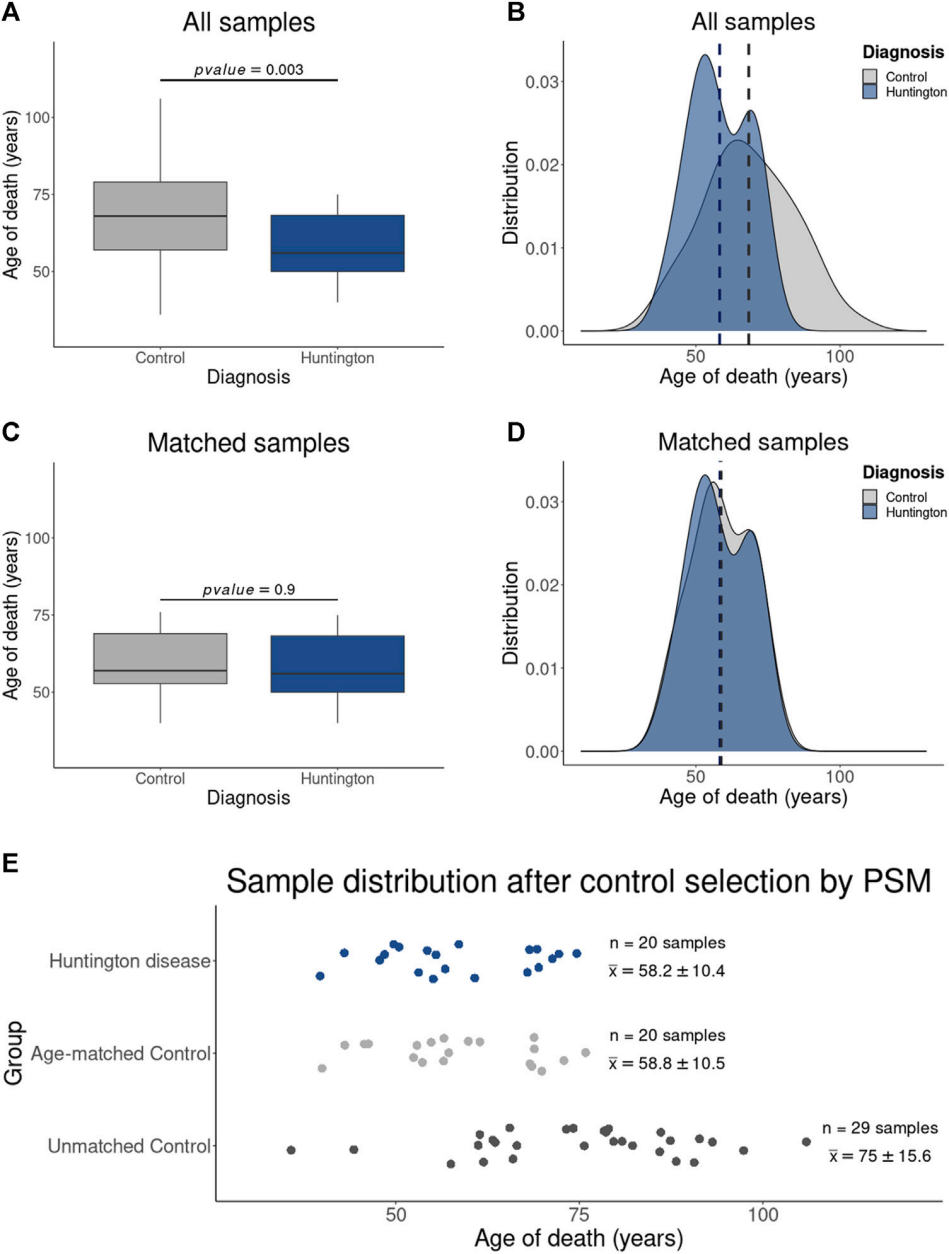
Results of Control Sample Selection Using PSM: The boxplot in **(A)** illustrates the statistically significant age difference between HD gene-positive individuals and the entire control sample set, as confirmed by the age distribution in **(B)**. Notably, **(C)** demonstrates the absence of a statistical difference in age between HD gene-positive individuals and the PSM-selected control samples, a finding corroborated by the corresponding age distribution in **(D)**. Additionally, the dispersion plot in I affirms that the excluded control samples (Unmatched controls) consist of older individuals (with an average age of 75 ± 15.6 years), diverging from both HD gene-positive individuals (58.2 ± 10.4 years) and PSM-selected age-matched controls (58.8 ± 10.5 years). **(E)** Graphic showing the samples included in this study were obtained from individual with HD and controls with similar age distribution. Samples from neurological normal individuals without age-matching were removed by the PSM.

As anticipated, the PSM method effectively selected control samples from neurologically normal individuals with a comparable age distribution to HD gene-positive individuals (Figure 2). Consequently, two distinct control groups were established: (i) the “All Controls” group, encompassing all neurologically normal individuals (*n* = 49, with an average age of 68.3 ± 15.8 years), replicating the control cohort employed in the study by Labadorf et al. (2015), and (ii) the “Age-Matched” group, formed by samples from neurologically normal individuals exhibiting a similar age distribution (*n* = 20, with an average age of 58.2 ± 10.4 years) to that of the HD gene-positive individuals (*n* = 20, with an average age of 58.8 ± 10.5 years), representing the “virtual” age-matched cohort.

### 3.3 Aging modifies the BA9 transcriptomic profile, making the older individuals non-appropriated controls for the DEG identification in Huntington’s disease

To assess the potential impact of utilizing control samples from older individuals on differential expression analysis, we initially compared the entire transcriptome of HD gene-positive individuals with that of all neurologically normal individuals using UMAP.

**FIGURE 3:**
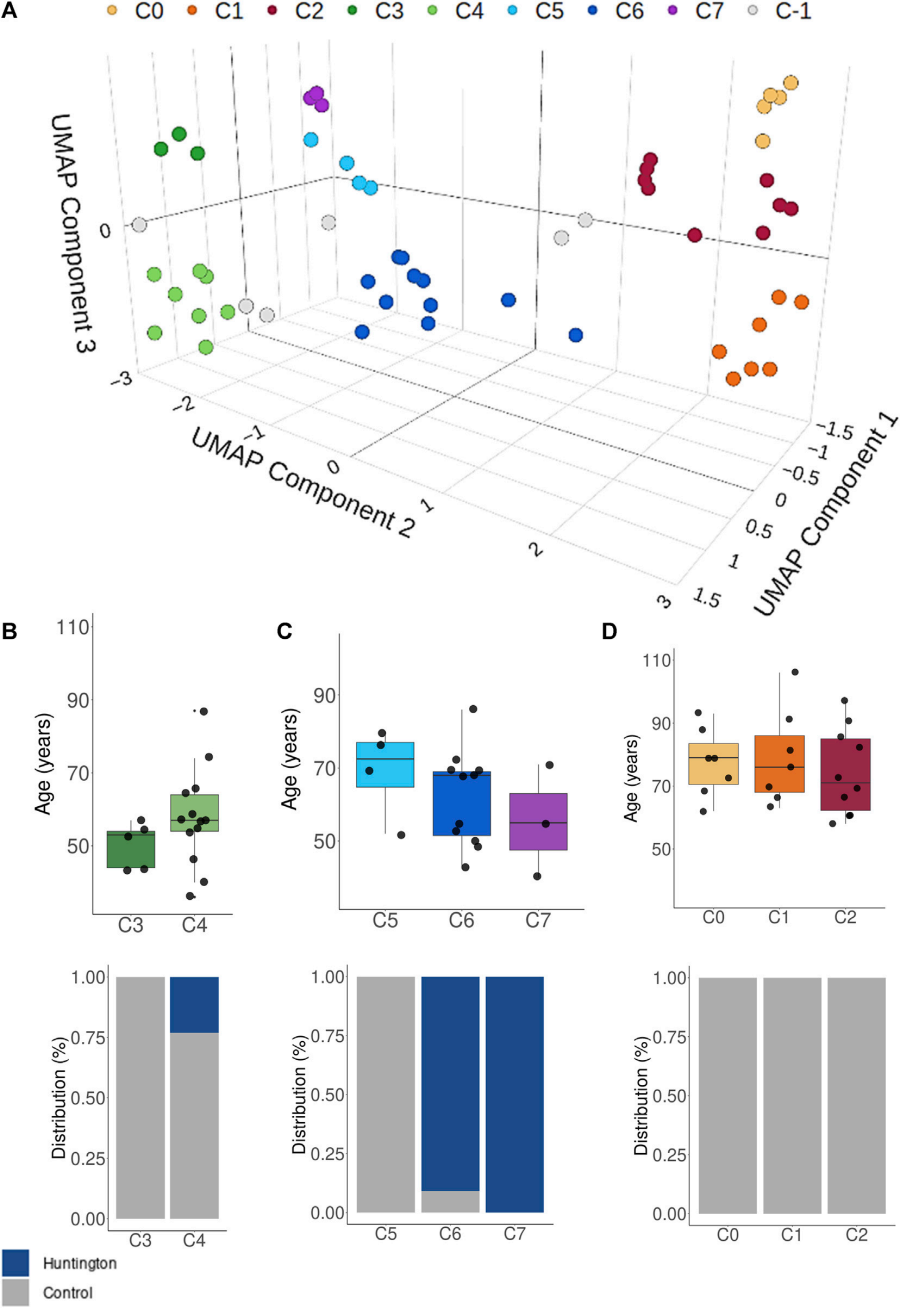
**(A)** UMAP and BDSCANclusterin analysis showing that the HD gene-positive individuals have mostly an intermediate overall transcriptomic expression between young controls and older controls. In the clustering analysis, C-1 (gray dots) represented unassigned samples to clusters based on their distance to the main groups. Plots **(B–D)** represents the age and condition distribution across all identified clusters. UMAP performed using all samples (*n* = 69).

The three UMAP-components effectively grouped most samples into seven distinct density-based clusters, revealing three overall patterns (Figure 3A). Notably, clusters C3 and C4 on the left side predominantly comprised young control individuals. In contrast, the transcriptome of HD gene-positive individuals (clusters C6 and C7) occupied an intermediary position on the Cartesian plane between neurologically normal young individuals and more distant older control individuals (aged over 70 years, Figures 3C,D). This outcome strongly suggests that age significantly influences the BA9 transcriptomic profile, emphasizing that using older individuals as controls may not be appropriate for identifying DEGs in HD.

### 3.4 Control samples from older individuals affect the differential expression analysis

**FIGURE 4:**
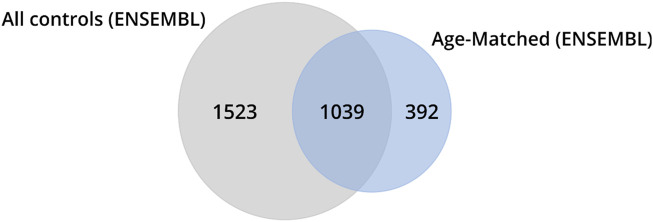
Venn diagrams illustrate the number of genes identified as DEGs in HD gene-positive individuals concerning all control samples. Among these, 1,523 were no longer reclassified as DEGs, 1,039 remained classified as DEGs, and 392 became differentially expressed after the exclusion of control samples from older individuals (age-matched controls).

To assess the impact of including older control individuals on the identification of DEGs, we conducted a comparison by analyzing the number of genes identified across various classes (ZCG, LCG, EEG, NSLFCG, URG, and DRG) in HD gene-positive individuals relative to (i) the entire control group (*n* = 49) and (ii) age-matched controls (*n* = 20) using a cross table. The results revealed that excluding older individuals from the control group led to the reclassification of 1,915 genes of interest. Among these, 1,523 (79.5%) were no longer considered DEGs (putative false-positive genes), while 392 (20.5%) transitioned to being classified as DEGs (putative false-negative genes) (Figure 4, highlighted in color in Table 3).

**TABLE 3:** Quantitative analysis of the impact of age of death-matched controls selection in terms of differential expression genes (DEG) classification. Numbers in black bold describe the total number of upregulated (URG) and/or downregulared genes (DRG).

			Age of death-matched controls						
			NDEG				DEG		
			ZCG	LCG	EEG	NSLFCG	URG	DRG	Total
All control	NDEG	ZCG	8,949	-	-	-	-	-	8,949
		LCG	-	30,412	28	-	7	-	30,447
		EEG	-	720	16,648	206	127	68	17,769
		NSLFCG	-	-	2014	772	121	69	2,976
	DEG	URG	-	13	582	14	711	-	**1,320**
		DRG	-	212	678	24	-	328	**1,242**
	Total		8,949	31,357	19,950	1,016	**966**	**465**	62,703

ZCG, zero count genes; LCG, low count genes; EEG, equally expressed genes; NSLFCG, non-significant log2 fold change genes; URG, upregulated genes; DRG, downregulated genes. Numbers highlighted in blue indicate genes that became DEG., Numbers highlighted in red indicate genes that were no longer DEG (or became NDEG). Numbers in bold describe the total number of upregulated (URG) and/or downregulared genes (DRG).

### 3.5 Incorporating samples from older control individuals has an impact on functional enrichment

**FIGURE 5:**
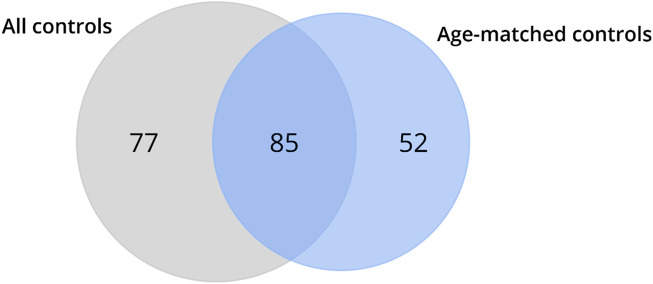
Venn diagrams depict the outcomes of the functional enrichment analysis using GSEA for biological pathways and molecular functions. The results demonstrate that the DEGs identified in HD gene-positive individuals using all control samples enriched for 77 biological pathways, which were no longer observed in the enrichment based on the DEGs identified using age-matched controls (denoted as excluded pathways). Conversely, the DEGs identified using age-matched controls enriched for 52 biological pathways that were not found in the analysis using all control samples (referred to as new pathways). A total of 85 biological pathways were enriched for the DEGs identified using both all and age-matched control samples (referred to as conserved pathways).

Recognizing that different genes can concurrently regulate multiple molecular functions in various biological processes, the DEGs identified in HD gene-positive individuals concerning all controls (*n* = 49) or age-matched controls (*n* = 20) were independently subjected to functional enrichment analysis using GSEA in terms of Geneontology (GO). The results revealed that protein-coding DEGs from all controls enriched for 162 biological pathways, while those obtained exclusively from age-matched controls enriched for 137 biological pathways (Figure 5; Supplementary Excel S2). Upon comparison, it was observed that 77 pathways (Supplementary Table S1) were no longer identified as enriched when samples from older neurologically normal individuals were excluded from the control group (Figure 5).

Among the pathways excluded is the one associated with cellular response to heat (Supplementary Table S1), encompassing heat shock genes previously reported as upregulated in HD^10–12^. Conversely, the omission of samples from older neurologically normal controls enriched 52 new pathways (Figure 5; Supplementary Table S2). A total of 85 pathways remained consistent between the analyses using all and age-matched controls (Figure 5; Supplementary Table S3). Interesting, we also observed that the genes that were no longer classified as DEG (highlighted in red in Table 3) are directly involved in inflammatory process (Supplementary Table S4), reinforcing that the aging exacerbates (neuro)inflammatory process which can lead to false-positive results. These findings underscore that the inclusion of samples from older control individuals not only leads to the misidentification of DEGs but also exerts a detrimental impact on functional enrichment.

### 3.6 The aging process amplifies the expression changes of both up- and downregulated genes in BA9, thereby adversely affecting the identification of HD-related DEGs

Considering the involvement of DEGs in HD pathophysiology, mediated by protein interactions within each biological pathway, we identified the protein-coding genes associated with pathways no longer enriched (235 genes, 77 excluded pathways) and those enriched in new pathways (222 genes, 52 pathways) identified in the analysis using age-matched controls. To assess the impact of aging on the expression of these genes, we categorized them based on their normalized counts into four groups reflecting their abundance levels (Table 4). This categorization facilitated the visualization of gene expression levels across age groups. We compared normalized counts of these genes (per category) between HD gene-positive and neurologically normal individuals aged less than 60, 60–75, and older than 75 years (the maximum age observed in the HD group, Figure 2). The rate estimates of expression changes across age in the control groups for the genes that became DEGs showed statistical significance.

**TABLE 4:** Count of genes (categorized as URG or DRG) that either became DEGs or were no longer classified as DEGs following age matching.

	Became DEG			No longer DEG		
Max count/gene	URG	DRG	Total	URP	DRG	Total
**<**900	94	12	106	118	28	146
900–3,000	55	3	58	35	9	44
3,000–13,000	35	8	43	27	10	37
**>**13,000 **^a^**	6	9	15	1	7	8
Total genes	190	32	222	181	54	235

^a^ Non graphically plotted due to the reduced number of genes belonging to this classification.

**FIGURE 6:**
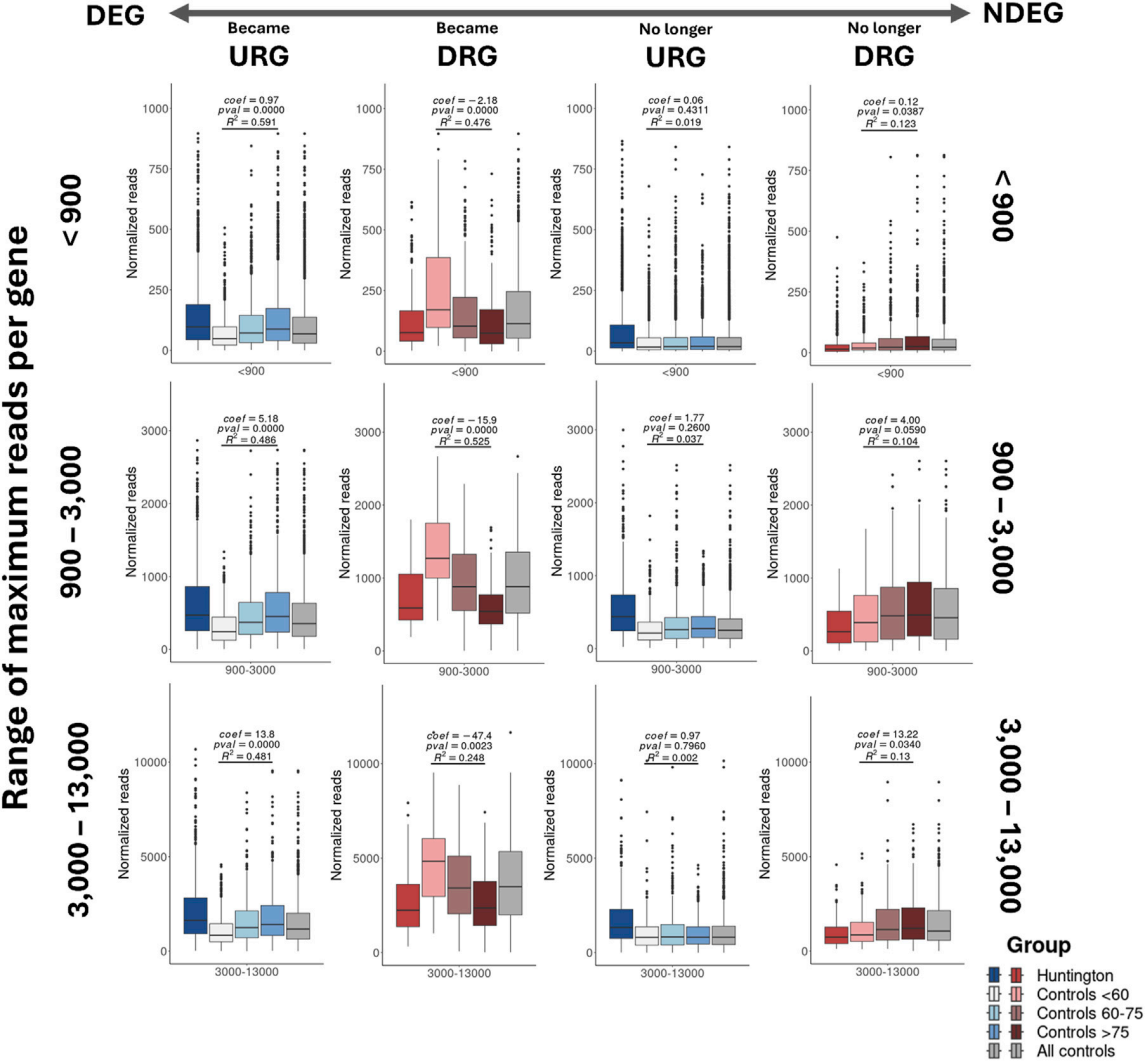
Analysis of the aging effect on protein-coding genes enriched for pathways revealed significant impacts on classification due to age-matching. The results indicate that aging has a more pronounced effect on genes that became DEGs, leading to a reduction in the expression levels of DRGs and an increase in the expression levels of URGs in HD gene-positive individuals compared to neurologically normal individuals aged less than 60 years. Additionally, the boxplots for all control samples display a median expression more closely resembling that of HD gene-positive individuals than those observed for neurologically normal individuals aged less than 60 years.

These results highlight that aging has a more pronounced effect on genes that became DEGs, reducing the expression levels of DRGs and increasing the expression levels of URGs in HD gene-positive individuals compared to neurologically normal controls aged under 60 years (Figure 6). Notably, the level of gene expression abundance correlates with the magnitude of changes observed across age groups.

These findings collectively affirm that aging significantly impacts gene expression in BA9. This underscores the importance of utilizing control samples with a comparable age distribution to HD gene-positive individuals, emphasizing that such an approach is preferable and imperative for accurately identifying DEGs with potential utility as pharmaceutical or prognostic targets. However, it’s worth noting that aging exhibits negligible effects on genes that are no longer URGs and have subtle effects on genes that are no longer DRGs (Figure 6).

### 3.7 Incorporating control samples from older neurologically normal individuals impacts *p*-value adjustment, thereby contributing to the false-positive identification of DEGs

**FIGURE 7:**
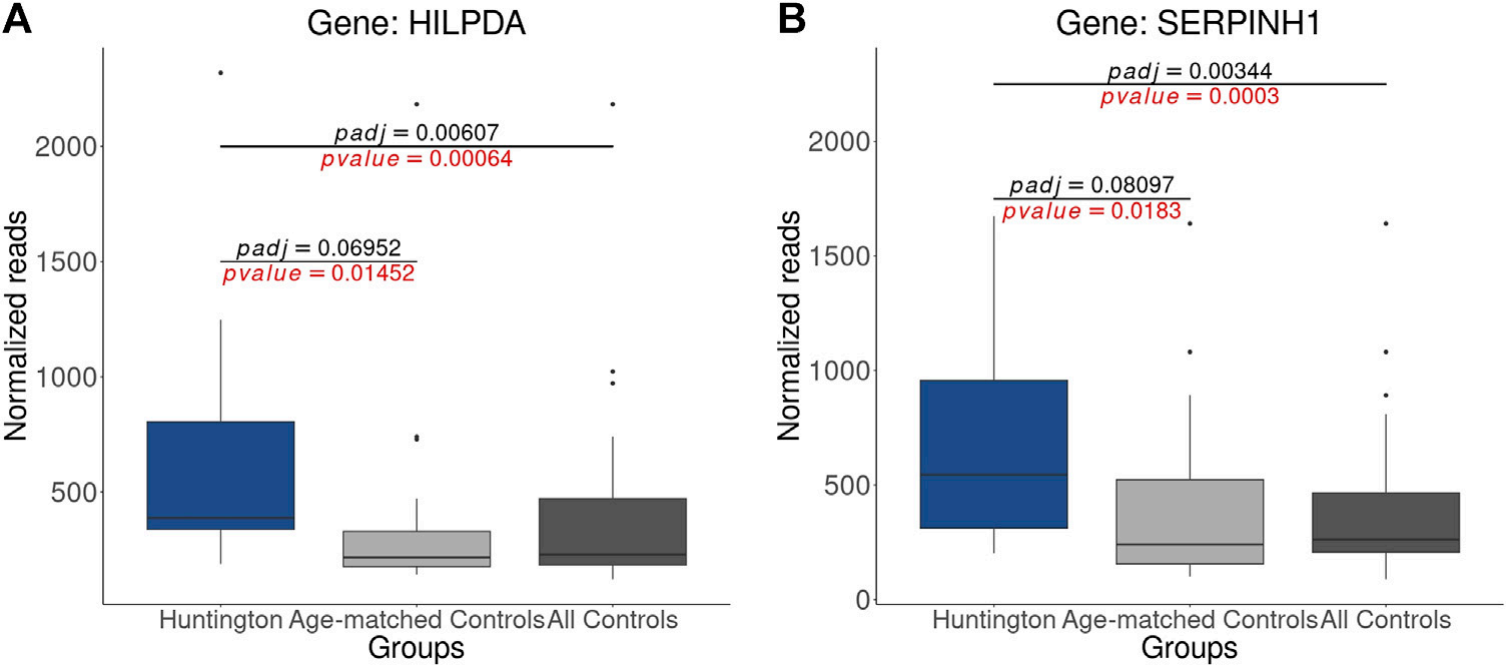
Statistical Disparities in Normalized Gene Counts for Genes Previously Identified as Upregulated in HD Gene-Positive Individuals: **(A)** HILPDA **(B)** SERPINH1. It is noteworthy that the *p*-values (highlighted in red) for both genes are significant (*p*-value < 0.05) in comparisons with both all controls and age-matched controls. However, when analyzing only age-matched controls, the adjusted *p*-values for both genes become non-significant (p-adjust > 0.05). This underscores the influence of age matching on the statistical outcomes and emphasizes its impact on the significance of gene expression changes.

The impact of age matching on *p*-value adjustment is evident in the reclassification of numerous DEGs to NDEGs (Supplementary Figure S1). To illustrate these findings, we examined the normalized counts of two genes (previously identified as upregulated by Labadorf et al. (2015)), *HILPDA* and *SERPINH1*, which were no longer classified as DEGs in the analysis with age-matched controls. The raw *p*-values obtained using both all controls and age-matched controls were statistically significant (*p*-value < 0.05). However, the *p*-value adjustment applied in DESeq2, which controls the false-discovery rate (FDR) and considers age-matched controls, resulted in a non-significant adjusted *p*-value (p-adjust > 0.05, Figure 7). This effect is observed in 3.206 genes (2.686 protein-coding genes), from which 478 genes had log2FC > 0.58 and 310 had log2FC < −0.58 (Supplementary Excel S3). These results underscore that the absence of age matching leads to the identification of false-positive DEGs due to *p*-value adjustment, reinforcing the findings in Figure 6.

Furthermore, we demonstrated that age matching also influences the log2 fold change (log2FC), altering the gene abundance levels of control samples, as depicted in Figure 6. As anticipated, this effect was more pronounced for genes that became DEGs and those that are no longer DEGs (Supplementary Figure S1) than those that remain classified as DEGs (Supplementary Figure S1). Collectively, these findings strongly support the contention that the lack of age matching leads to the misidentification of DEGs.

### 3.8 The synergy of age matching with an analytical strategy can foster the discovery of pharmaceutical targets

**FIGURE 8:**
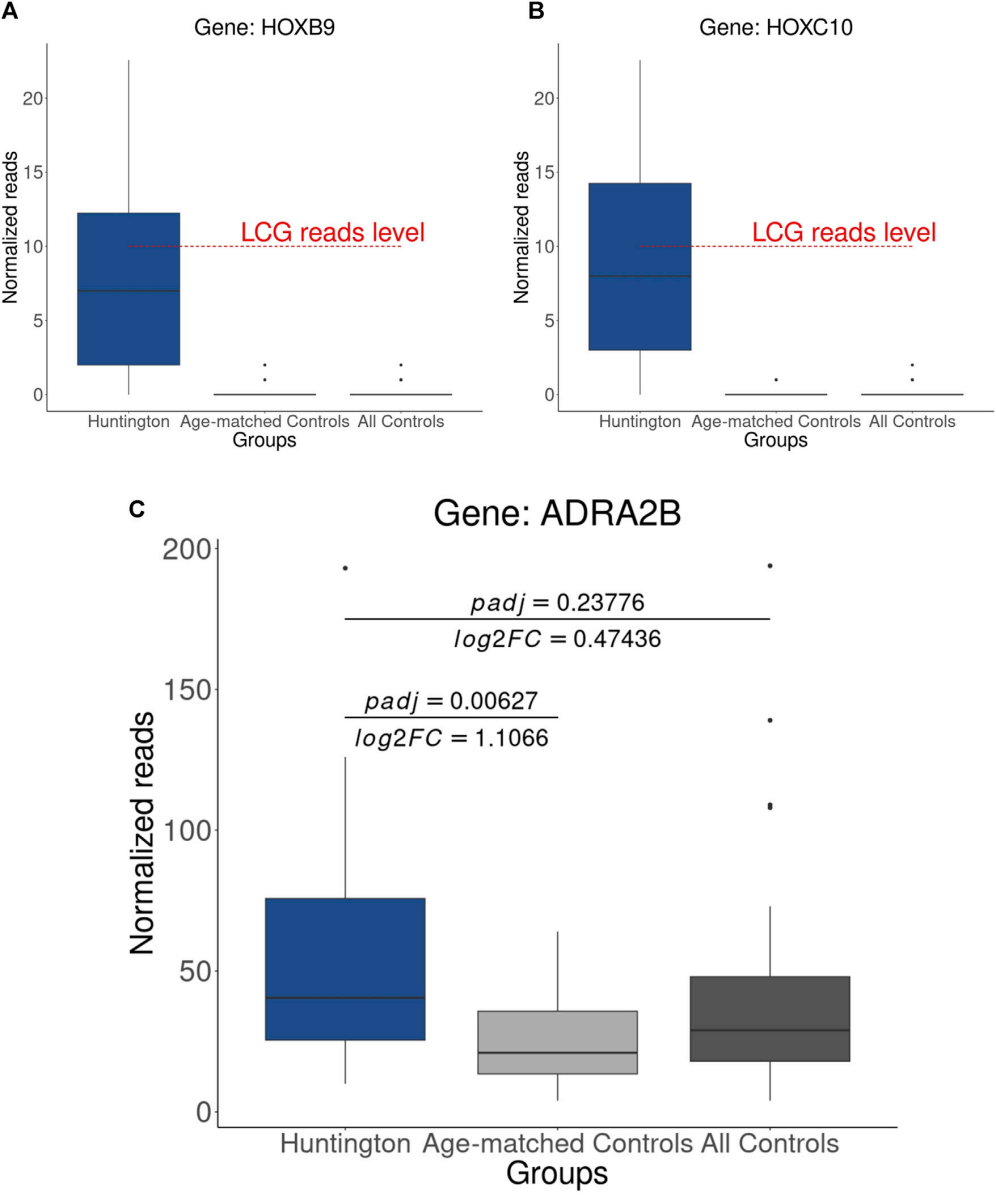
Expression Levels of Heat Shock Protein-Coding Genes: **(A)** HOXB9, **(B)** HOXC10 These genes were previously identified as upregulated, and it is noteworthy that the median of normalized counts in HD gene-positive individuals is below the threshold typically considered for gene expression. **(C)** Upregulated gene (ADRA2B) identified using age-matched controls, a result obtained through the combination of adjusted *p*-value, log2FC, and normalized counts.

In our final assessment, we examined the expression levels, based on normalized counts, of genes previously identified as URGs in HD gene-positive individuals by Labadorf et al. (2015), using both all controls and age-matched controls. Interestingly, we noted that the heat shock protein-coding genes identified as URGs in HD by Labadorf et al. (2015) exhibit relatively low expression, with a median expression in HD gene-positive individuals falling below the commonly used threshold for defining gene expression (count > 10, Figures 8A,B). This observation suggests that, in addition to age matching in the control group, normalized counts should be further scrutinized with adjusted *p*-values and log2FC for accurate DEG identification.

**FIGURE 9:**
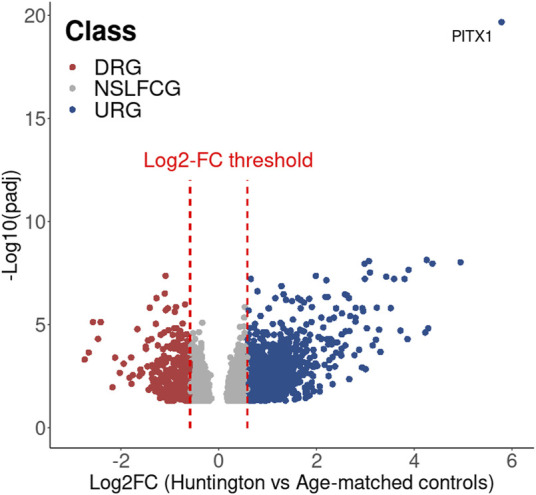
Volcano plot showing the DEG identified in HD in relation to the age-matched controls.

Consequently, through the integration of adjusted *p*-values, log2FC, and normalized counts, we identified *ADRA2B* as upregulated in HD gene-positive individuals (Figure 8C), thereby emerging as a potential candidate for HD treatment. This underscores the importance of a comprehensive analytical approach for robust and accurate DEG identification. The genes identified as putative DEGs in HD (in relation to age-matched controls) can be visualized in Figure 9; Supplementary Excel S3.

## 4 Discussion

Despite the elucidation of the genetic basis of HD by MacDonald et al., in 1993, there is currently no approved treatment capable of altering the natural progression of the disease. To identify potential DEGs that could serve as pharmaceutical and/or prognostic targets for HD, various studies have conducted comparative analyses of the transcriptome across different brain areas from HD gene-positive and neurologically normal individuals (Labadorf et al., 2015; Agus et al., 2019). Although these studies adhered to technical recommendations to ensure optimal practices in RNA-Seq analysis (Conesa et al., 2016; Chung et al., 2021), none of the genes identified as upregulated by these investigations have yet proven successful as prognostic or therapeutic targets for HD.

Upon scrutinizing one of these studies, we noticed that the control group used to identify differentially expressed genes (DEGs) consisted of samples from neurologically normal individuals older than the HD gene-positive group (Labadorf et al., 2015). Considering that aging induces gradual transcriptional changes in the brain, leading to proteotoxic stress and iron accumulation, which can contribute to neuroinflammation (Hagemeier et al., 2012; González-Velasco et al., 2020; Ham and Lee, 2020) and result in motor and cognitive declines (Ham and Lee, 2020), a phenomenon also observed in HD (Domínguez et al., 2016; Barron et al., 2021; Jia et al., 2022; van de Zande et al., 2023; Wilton et al., 2023), we hypothesized that utilizing samples from older controls might lead to misidentification of DEGs. To validate our hypothesis, we reanalyzed the 69 samples previously investigated in this study (Labadorf et al., 2015), which are accessible on the SRA database. PSM, we formed a “virtual” control group comprising samples from neurologically normal individuals with a statistically similar age distribution to the HD gene-positive group.

To comprehensively visualize the entire transcriptome of the 69 analyzed samples, we employed unsupervised dimensionality reduction combined with density-based clustering techniques. The results revealed distinct clusters, each exhibiting characteristic age-related features. As anticipated, the 20 samples from HD gene-positive individuals differed from the control samples. However, the transcriptome of HD gene-positive individuals demonstrated a transitional state, aligning more closely with neurologically normal individuals aged over 70 and younger controls under 70. These findings provide evidence supporting the notion that Huntington’s disease accelerates biological aging in the brain, as previously discussed in the literature (Horvath et al., 2016; Machiela and Southwell, 2020; Alcalá-Vida et al., 2021).

**FIGURE 10:**
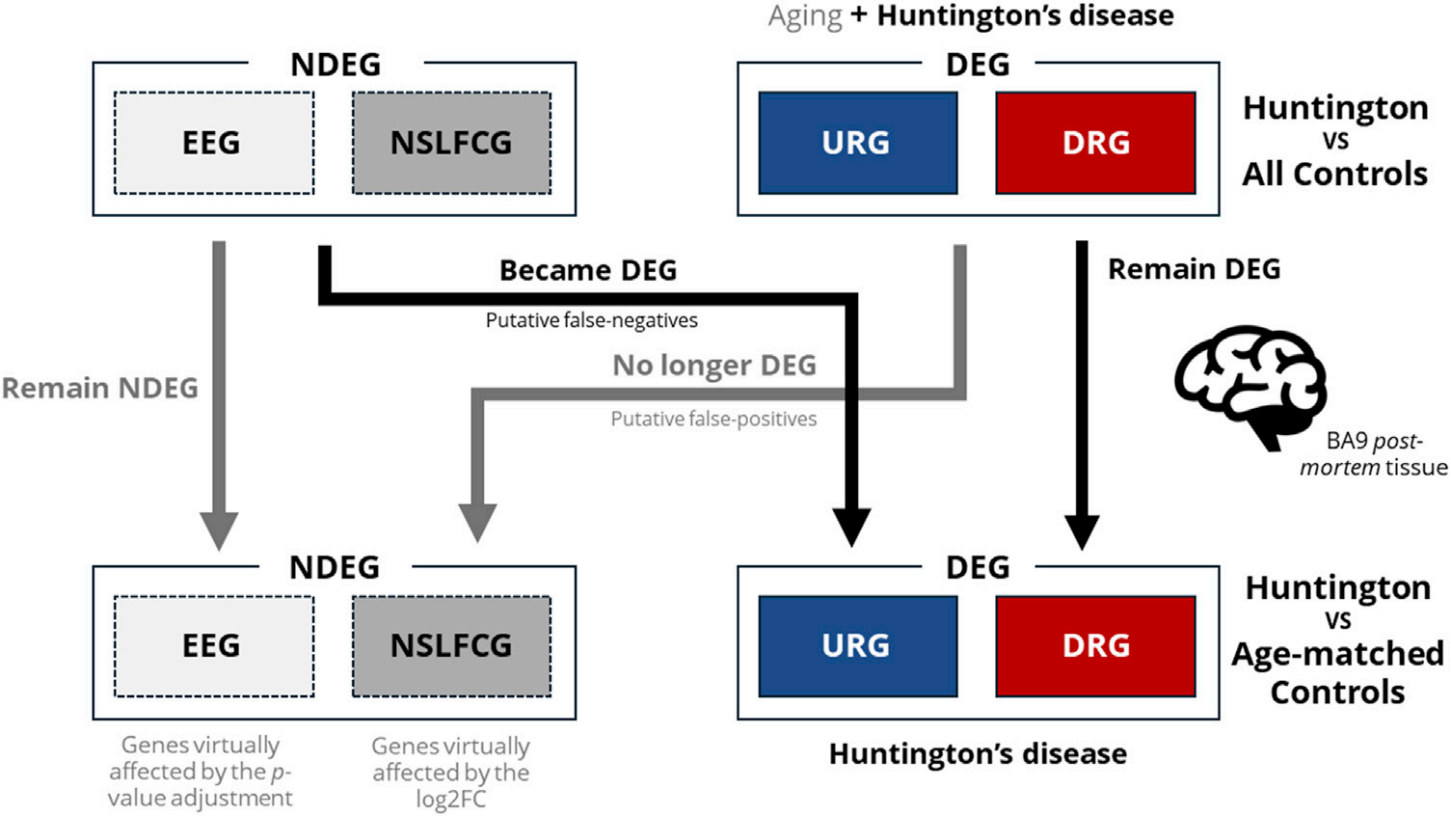
Illustration of how the utilization of control samples from older neurologically normal individuals can impact accurate DEGs identification. As observed, age matching (via PSM) resulted in the transition of NDEGs to DEGs, highlighting the negative impact of using control samples from older neurologically normal individuals on DEG discovery and increasing the likelihood of type II errors (false negatives). Additionally, age matching led to the reclassification of DEGs to NDEGs through *p*-value adjustment (reclassifying DEGs as equally expressed genes–EEG) or significantly affected log2FC (reclassifying DEGs as non-significant log2FC genes–NSLFCG). These findings provide evidence that the use of older neurologically normal individuals as controls also elevates the probability of type I errors (false positives).

To evaluate the impact of using samples from older neurologically normal controls on differential expression analysis (DEA), we compared the BA9 transcriptome of HD gene-positive individuals (*n* = 20, 58.8 ± 10.5 years) with two groups: all controls (*n* = 49, 68.3 ± 15.8 years) and age-matched controls (*n* = 20, 58.2 ± 10.4 years). We categorized genes into six classes based on expression and differentiation levels. Our findings illustrate that age-matching influences the identification of differentially expressed genes (DEGs) in distinct ways, as depicted in Figure 10.

The age matching of the control group not only significantly reduced the number of Differentially Expressed Genes (DEGs) from 2,562 to 1,431 by eliminating putative false-positive DEGs but also facilitated the identification of novel putative DEGs. Consequently, it is unsurprising that the analysis based on the “virtual” control group also impacted the functional enrichment analyses. This led to the exclusion of 77 enriched biological pathways and the emergence of 52 new biological pathways.

Remarkably, among the pathways excluded was the one related to the cellular response to heat, encompassing heat shock protein-coding genes previously identified as upregulated in HD by Labadorf et al. (2015). Analyzing the normalized reads of these genes, we observed that they have a median expression below the reliable threshold of detection in the HD gene-positive group. However, despite not being expressed in the control group, these HOX genes exhibit a significant log2FC, providing a rationale for the results obtained by Labadorf et al. (2015).

We also observed that aging primarily impacts the expression of genes that became DEGs, leading to a gradual increase in the expression of URGs and a decrease in the expression of DRGs. This shift consequently alters the log2FC between HD and controls. This finding aligns with existing literature, where evidence indicates that aging induces a progressive deterioration of physiological and biochemical functions in the brain (Lupo et al., 2019; Ham and Lee, 2020), promoting transcriptional changes in BA9.

Differential expression analysis using DESeq2 revealed the lack of significant differences (*p*-value > 0.05) for numerous enriched genes when comparing HD gene-positive individuals with neurologically normal individuals, including those aged older than 75 years. In contrast, when age-matching was performed, significant statistical differences (*p*-value < 0.05) were observed for the same genes. Collectively, these findings strongly indicate that the inappropriate selection of samples from neurologically normal controls increases the likelihood of type II errors (false negatives), leading to the oversight of potential DEGs.

**FIGURE 11:**
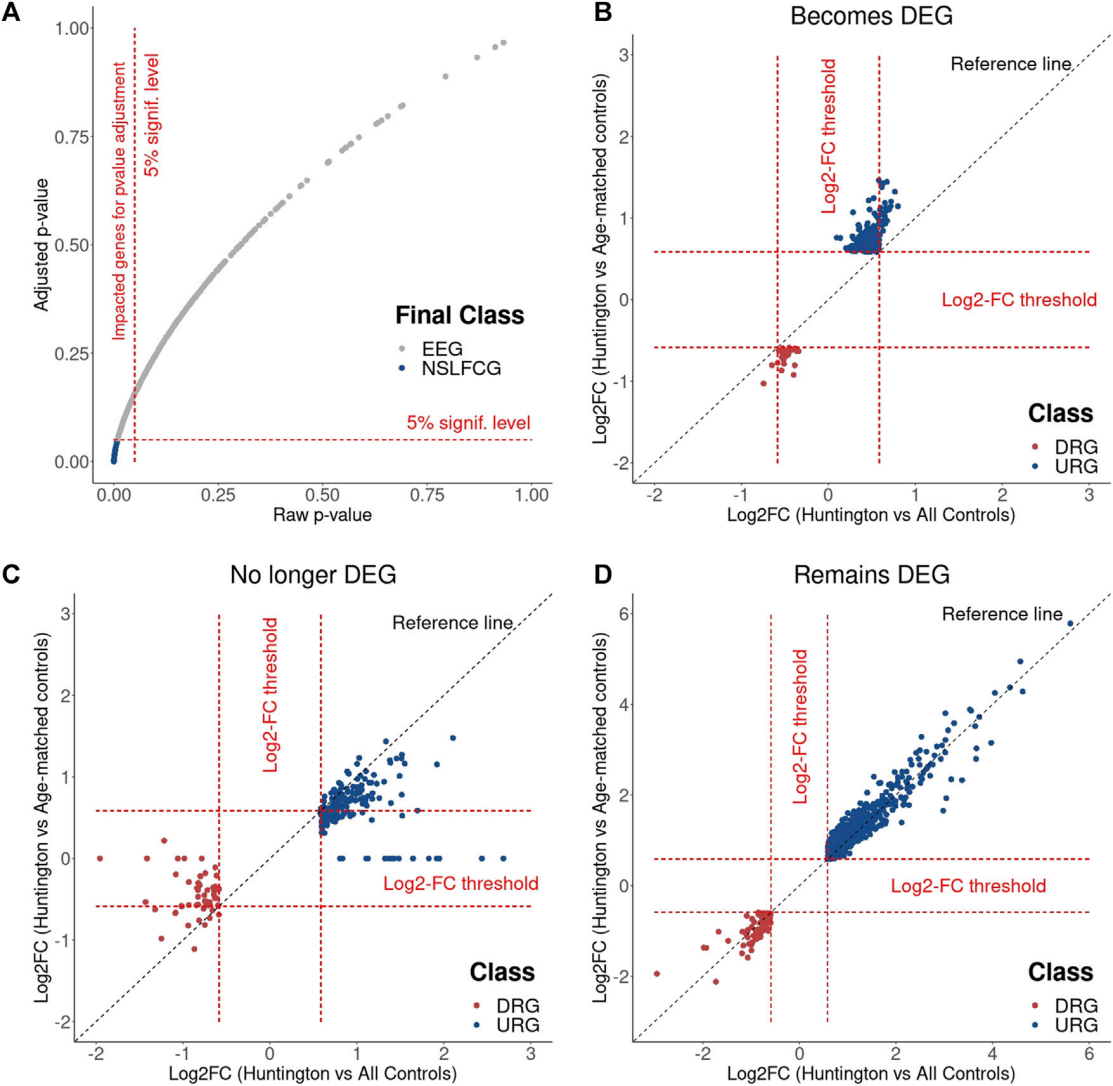
Effects of the age matching on differential expression analysis. **(A)** Results show that the age matching affects the *p*-value adjustment, reclassifying part of DEGs to NDEGs. Log2FC changes observed in genes that became DEG **(B)**, that were no longer DEG **(C)** and that remain DEG **(D)** with the age matching.

Differential expression analysis based on DESeq2 revealed no statistical differences among the enriched genes no longer classified as DEGs. This suggests that the inclusion of older neurologically normal controls indirectly contributes to type I errors, leading to putative false-positive DEGs. This hypothesis is plausible because the raw *p*-values obtained from the Wald test (utilized for DEG identification by DESeq2) are corrected for multiple testing using the Benjamin and Hochberg (BH) method to control the false-discovery rate (FDR) (Benjamin and Hochberg, 1995). Consequently, removing older neurologically normal individuals from the “virtual” control group altered the raw *p*-values of many genes, influencing the adjusted *p*-values (Figure 11).

To illustrate this, we analyzed the normalized counts of two genes identified as upregulated in HD by Labadorf et al. (2015) (*HILPDA* and *SERPINH1*) in HD gene-positive individuals, age-matched controls, and all controls (including older neurologically normal individuals). During the differential analysis, the Wald test revealed a statistically significant difference (raw *p*-value < 0.05) in both scenarios during the differential analysis. However, the *p*-value adjustment for these genes became nonsignificant (adjusted *p*-value > 0.05) after removing older neurologically normal individuals, leading to the false-positive discovery of DEGs.

Considering that identifying pharmaceutical/prognostic targets often relies on selecting disease phenotype-related genes ranked with higher log2FC, these findings underscore the impact of using inappropriate control samples from neurologically normal individuals, leading to misguidance in DEG identification. By excluding neurologically normal individuals older than 70 years, we identified a novel putative therapeutic target, the ADRA2B gene, encoding the alpha-2 adrenergic receptor. This gene is upregulated in HD and is a target for various approved antipsychotic drugs, including levomepromazine, pramipexole, ropinirole, aripiprazole, ziprasidone, promazine, and nortriptyline (Huang et al., 2022). Moreover, *in vitro* treatment with beditin, a novel alpha-2 adrenoreceptor antagonist, has demonstrated a significant cytotoxicity reduction, increasing neuronal cell survival (Singer et al., 2021). These findings position the alpha-2 adrenoreceptor as a potential pharmaceutical target for HD, with beditin being a promising candidate for pharmaceutical receptor manipulation. However, further preclinical, and clinical studies are essential to confirm the therapeutic potential of beditin.

It's worth noting that this study is part of a broader investigation dedicated to analyzing RNA-Seq data for HD. Our upcoming study will present candidate genes for pharmaceutical/prognostic targets, integrating different BioProjects and analytical strategies, including artificial intelligence, to identify potential DEGs for drug discovery and development accurately.

## 5 Conclusion

In conclusion, our study strongly indicates that employing neurologically normal individuals aged over 70 as controls has a detrimental impact on the accuracy of differential expression analysis, leading to both false-positive and false-negative Differentially Expressed Genes (DEGs). While the focus of this study is on Huntington’s disease, our results imply that the thoughtful inclusion of age-appropriate control samples in study design can significantly enhance the best practices of differential expression analysis. This study also suggests that the lack of demographic feature matching between cases and controls, such as sex, can lead to DEG misidentification.

## Data Availability

The datasets presented in this study can be found in online repositories. The names of the repository/repositories and accession number(s) can be found in the article/Supplementary Material.
