# Supplementary figures and images for: How does the age of control individuals hinder the identification of target genes for Huntington’s disease?
Source: Front Genet. 2024 Jun 20;15:1377237. doi: 10.3389/fgene.2024.1377237 (PMC11228582; doi:10.3389/fgene.2024.1377237)

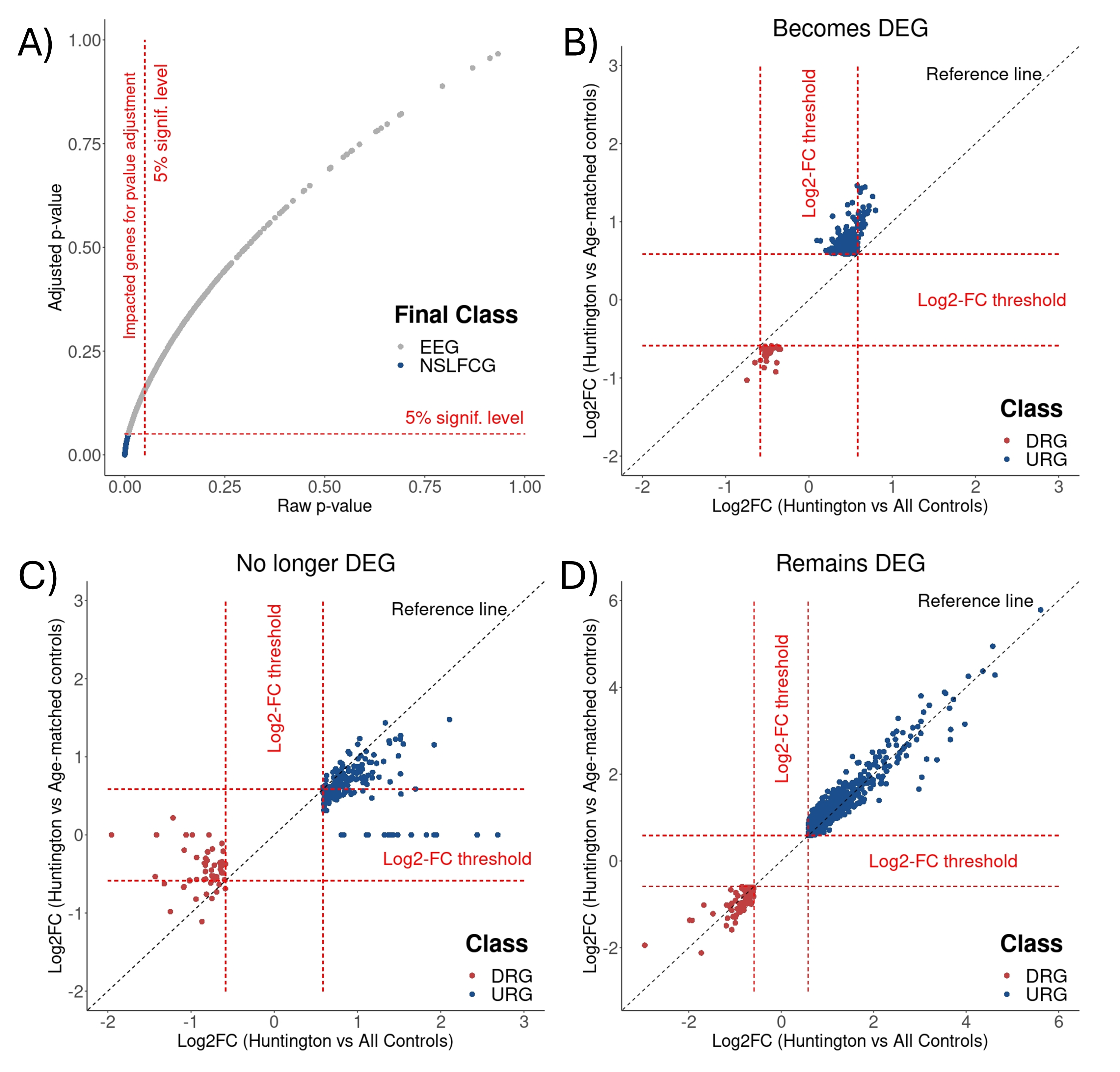

Supplement: Supplementary file 3 [file Image1.TIF]
