# Supplementary material for: How does the age of control individuals hinder the identification of target genes for Huntington’s disease?
Source: Front Genet. 2024 Jun 20;15:1377237. doi: 10.3389/fgene.2024.1377237 (PMC11228582; doi:10.3389/fgene.2024.1377237)
Supplement: Supplementary file 5 [file Table4.DOCX]

Supplementary Material

# Supplementary Figures and Tables

**Suplemmentary table 1**. Biological pathways exclusively enriched in the analysis using all control samples.

| Pathway | FDR | NES | URP/DRP |
| --- | --- | --- | --- |
| Myeloid leukocyte migration | 0.0000 | 2.8853 | URP |
| Granulocyte chemotaxis | 0.0000 | 2.7118 | URP |
| Cell chemotaxis | 0.0000 | 2.6098 | URP |
| Positive regulation of immune system process | 0.0000 | 2.5283 | URP |
| Wound healing | 0.0000 | 2.4865 | URP |
| Cytokine mediated signaling pathway | 0.0000 | 2.4305 | URP |
| Cytokine binding | 0.0001 | 2.4267 | URP |
| Embryonic hindlimb morphogenesis | 0.0000 | 2.4010 | URP |
| Cellular response to lipid | 0.0000 | 2.3966 | URP |
| Leukocyte differentiation | 0.0000 | 2.3899 | URP |
| Appendage morphogenesis | 0.0000 | 2.3724 | URP |
| Ossification | 0.0000 | 2.3481 | URP |
| Regulation of cell adhesion | 0.0000 | 2.3401 | URP |
| Embryonic forelimb morphogenesis | 0.0001 | 2.3213 | URP |
| Negative regulation of peptidase activity | 0.0000 | 2.3155 | URP |
| T cell activation involved in immune response | 0.0001 | 2.3142 | URP |
| Vascular transport | 0.0002 | 2.3065 | URP |
| Odontogenesis | 0.0001 | 2.2913 | URP |
| Response to cytokine | 0.0000 | 2.2889 | URP |
| Embryo development | 0.0000 | 2.2869 | URP |
| Mesenchyme development | 0.0000 | 2.2819 | URP |
| Sensory organ development | 0.0000 | 2.2746 | URP |
| Mesenchymal cell proliferation | 0.0005 | 2.2740 | URP |
| Cranial skeletal system development | 0.0002 | 2.2669 | URP |
| Regulation of response to stress | 0.0000 | 2.2564 | URP |
| Muscle tissue development | 0.0000 | 2.2303 | URP |
| Pattern specification process | 0.0000 | 2.2291 | URP |
| Positive regulation of developmental process | 0.0000 | 2.2215 | URP |
| Tissue migration | 0.0000 | 2.2151 | URP |
| Cytokine activity | 0.0010 | 2.1928 | URP |
| B cell receptor signaling pathway | 0.0006 | 2.1904 | URP |
| Locomotion | 0.0000 | 2.1774 | URP |
| Organ growth | 0.0004 | 2.1577 | URP |
| Circulatory system process | 0.0000 | 2.1509 | URP |
| Regulation of cell death | 0.0000 | 2.1207 | URP |
| Organic anion transmembrane transporter activity | 0.0017 | 2.0989 | URP |
| Organic acid transport | 0.0003 | 2.0923 | URP |
| Erk1 and Erk2 cascade | 0.0003 | 2.0770 | URP |
| G protein coupled chemoattractant receptor activity | 0.0006 | 2.0667 | URP |
| Epithelial cell apoptotic process | 0.0024 | 2.0567 | URP |
| Collagen binding | 0.0116 | 2.0476 | URP |
| Smooth muscle tissue development | 0.0003 | 2.0463 | URP |
| Multi multicellular organism process | 0.0028 | 2.0408 | URP |
| Digestive system development | 0.0016 | 2.0406 | URP |
| Response to oxygen containing compound | 0.0000 | 2.0193 | URP |
| Positive regulation of phospholipase activity | 0.0075 | 2.0138 | URP |
| WW domain binding | 0.0032 | 1.9903 | URP |
| Angiogenesis involved in wound healing | 0.0129 | 1.9895 | URP |
| Muscle structure development | 0.0001 | 1.9884 | URP |
| Response to prostaglandin | 0.0122 | 1.9790 | URP |
| Positive regulation of cell communication | 0.0000 | 1.9712 | URP |
| Cellular response to heat | 0.0162 | 1.9492 | URP |
| Cellular response to ketone | 0.0115 | 1.9482 | URP |
| Cell adhesion molecule binding | 0.0006 | 1.9456 | URP |
| Negative regulation of response to stimulus | 0.0000 | 1.9396 | URP |
| Positive regulation of epithelial cell proliferation involved in wound healing | 0.0011 | 1.9368 | URP |
| Ureter development | 0.0129 | 1.9332 | URP |
| Negative regulation of small molecule metabolic process | 0.0183 | 1.9137 | URP |
| Platelet derived growth factor binding | 0.0233 | 1.8860 | URP |
| Skin morphogenesis | 0.0064 | 1.8555 | URP |
| Protein phosphorylated amino acid binding | 0.0461 | 1.8455 | URP |
| Icosanoid receptor activity | 0.0269 | 1.8209 | URP |
| Receptor mediated endocytosis | 0.0125 | 1.8171 | URP |
| Transmembrane receptor protein serine threonine kinase binding | 0.0225 | 1.8100 | URP |
| Desmosome organization | 0.0256 | 1.8043 | URP |
| IgG binding | 0.0258 | 1.7868 | URP |
| G protein coupled receptor signaling pathway | 0.0011 | 1.7781 | URP |
| Negative regulation of biosynthetic process | 0.0001 | 1.7595 | URP |
| Molecular function regulator activity | 0.0010 | 1.6430 | URP |
| Monoatomic cation transmembrane transporter activity | 0.0457 | 1.5381 | DRP |
| Microtubule based movement | 0.0193 | 1.7316 | DRP |
| Negative regulation of microtubule polymerization or depolymerization | 0.0473 | 1.8405 | DRP |
| G protein coupled amine receptor activity | 0.0345 | 1.9031 | DRP |
| Detection of abiotic stimulus | 0.0195 | 1.9211 | DRP |
| Catechol containing compound metabolic process | 0.0261 | 1.9271 | DRP |
| Serotonin receptor signaling pathway | 0.0084 | 1.9420 | DRP |
| Cytoskeletal motor activity | 0.0072 | 2.1561 | DRP |

URP – upregulated pathway; DRP – downregulated pathway. Pathways ranked according to the normalized enrichment score (NES). Pathways highlighted in grey correspond to biological pathways not related to the HD. Pathway highlighted in blue indicate biological pathway that contains the target genes identified in previous published studies.

**Suplemmentary table 2**. Novel biological pathways identified as consequence of the novel DEGs identified using age-matched samples

| Pathway | FDR | NES | URP/DRP |
| --- | --- | --- | --- |
| Defense response to bacterium | 0.0000 | 2.378644 | URP |
| Mesoderm development | 0.0000 | 2.324034 | URP |
| Myeloid leukocyte activation | 0.0000 | 2.312897 | URP |
| Leukocyte chemotaxis | 0.0001 | 2.249987 | URP |
| Cellular response to zinc ion | 0.0001 | 2.246592 | URP |
| Leukocyte migration | 0.0000 | 2.212649 | URP |
| Cellular response to copper ion | 0.0002 | 2.201082 | URP |
| Positive regulation of cytokine production | 0.0000 | 2.177617 | URP |
| Vascular process in circulatory system | 0.0001 | 2.164396 | URP |
| Interleukin 10 production | 0.0005 | 2.159452 | URP |
| Response to wounding | 0.0000 | 2.14723 | URP |
| Epithelial cell differentiation involved in kidney development | 0.0011 | 2.141497 | URP |
| Monocarboxylic acid transport | 0.0015 | 2.075178 | URP |
| Inositol lipid mediated signaling | 0.0011 | 2.065979 | URP |
| Collagen metabolic process | 0.0025 | 2.052255 | URP |
| Antigen processing and presentation of peptide or polysaccharide antigen via mhc class ii | 0.0028 | 2.020745 | URP |
| Regulation of stress activated protein kinase signaling cascade | 0.0030 | 1.991474 | URP |
| Reactive oxygen species metabolic process | 0.0042 | 1.947153 | URP |
| Sulfur compound transmembrane transporter activity | 0.0268 | 1.918391 | URP |
| Immunoglobulin binding | 0.0152 | 1.879315 | URP |
| Antimicrobial humoral response | 0.0179 | 1.873664 | URP |
| Fatty acid binding | 0.0268 | 1.869912 | URP |
| Cellular response to alcohol | 0.0248 | 1.858268 | URP |
| Aminoglycan metabolic process | 0.0187 | 1.855691 | URP |
| Heterotypic cell cell adhesion | 0.0252 | 1.854513 | URP |
| Apoptotic process | 0.0000 | 1.838259 | URP |
| Regulation of protein maturation | 0.0248 | 1.83624 | URP |
| Cellular response to acid chemical | 0.0406 | 1.792581 | URP |
| Receptor serine threonine kinase binding | 0.0373 | 1.783978 | URP |
| Negative regulation of gene expression | 0.0001 | 1.779101 | URP |
| Lipid transporter activity | 0.0446 | 1.777834 | URP |
| Embryonic placenta morphogenesis | 0.0193 | 1.759974 | URP |
| Developmental process involved in reproduction | 0.0028 | 1.744627 | URP |
| Positive regulation of intracellular signal transduction | 0.0005 | 1.731293 | URP |
| Sequence specific dna binding | 0.0065 | 1.574531 | URP |
| Cytoskeleton dependent intracellular transport | 0.0267 | -1.817 | DRP |
| Trna metabolic process | 0.0430 | -1.87793 | DRP |
| Neuron cellular homeostasis | 0.0459 | -1.92094 | DRP |
| Amino acid catabolic process | 0.0476 | -1.93133 | DRP |
| Excitatory extracellular ligand gated monoatomic ion channel activity | 0.0436 | -1.95431 | DRP |
| Positive regulation of potassium ion transport | 0.0430 | -1.95602 | DRP |
| Neurotransmitter receptor internalization | 0.0277 | -2.04589 | DRP |
| Regulation of short term neuronal synaptic plasticity | 0.0109 | -2.04621 | DRP |
| Associative learning | 0.0267 | -2.07561 | DRP |
| Keratinization | 0.0150 | -2.07939 | DRP |
| Monoatomic cation channel activity | 0.0030 | -2.08575 | DRP |
| Neuropeptide receptor activity | 0.0268 | -2.11113 | DRP |
| Membrane depolarization during action potential | 0.0089 | -2.18352 | DRP |
| Dopamine secretion | 0.0042 | -2.35317 | DRP |
| Neuropeptide signaling pathway | 0.0004 | -2.57925 | DRP |
| Synaptic vesicle exocytosis | 0.0000 | -2.96216 | DRP |
| Vesicle mediated transport in synapse | 0.0000 | -3.10948 | DRP |

URP – upregulated pathway; DRP – downregulated pathway

**Suplemmentary table 3**. Biological pathways commonly enriched based on the DEGs identified using all controls and age-matched controls

| Pathway | FDR | NES | URP/DRP |
| --- | --- | --- | --- |
| Inflammatory response | 0.0000 | 2.805981 | URP |
| Response to bacterium | 0.0000 | 2.771082 | URP |
| Neutrophil migration | 0.0000 | 2.733392 | URP |
| Immune response | 0.0000 | 2.713282 | URP |
| Defense response | 0.0000 | 2.704877 | URP |
| Vasculature development | 0.0000 | 2.633313 | URP |
| Tube morphogenesis | 0.0000 | 2.616332 | URP |
| Cell activation | 0.0000 | 2.577516 | URP |
| Immune receptor activity | 0.0000 | 2.568267 | URP |
| Tissue morphogenesis | 0.0000 | 2.567917 | URP |
| Humoral immune response | 0.0000 | 2.543262 | URP |
| Epithelial cell proliferation | 0.0000 | 2.542329 | URP |
| Embryonic morphogenesis | 0.0000 | 2.53155 | URP |
| Regulation of immune system process | 0.0000 | 2.520602 | URP |
| Biological process involved in interspecies interaction between organisms | 0.0000 | 2.511541 | URP |
| Skeletal system development | 0.0000 | 2.509114 | URP |
| Regulation of response to external stimulus | 0.0000 | 2.472325 | URP |
| Circulatory system development | 0.0000 | 2.450481 | URP |
| Regulation of epithelial cell differentiation | 0.0000 | 2.441332 | URP |
| Embryonic skeletal system morphogenesis | 0.0000 | 2.436574 | URP |
| Anatomical structure formation involved in morphogenesis | 0.0000 | 2.424535 | URP |
| Granulocyte activation | 0.0000 | 2.40379 | URP |
| Interleukin 8 production | 0.0000 | 2.401546 | URP |
| Animal organ morphogenesis | 0.0000 | 2.396798 | URP |
| Regulation of multicellular organismal development | 0.0000 | 2.392654 | URP |
| Peptidase regulator activity | 0.0000 | 2.390352 | URP |
| Ear development | 0.0000 | 2.390176 | URP |
| Tissue remodeling | 0.0000 | 2.34756 | URP |
| Integrin binding | 0.0001 | 2.34699 | URP |
| Cytokine production | 0.0000 | 2.341859 | URP |
| Regulation of cell population proliferation | 0.0000 | 2.341518 | URP |
| Endocrine system development | 0.0001 | 2.340607 | URP |
| Signaling receptor binding | 0.0000 | 2.339285 | URP |
| Muscle cell proliferation | 0.0000 | 2.326896 | URP |
| Positive regulation of multicellular organismal process | 0.0000 | 2.324945 | URP |
| Regulation of peptidase activity | 0.0000 | 2.317012 | URP |
| Epithelium development | 0.0000 | 2.31451 | URP |
| Cell killing | 0.0001 | 2.312574 | URP |
| Modified amino acid transport | 0.0002 | 2.304009 | URP |
| Phagocytosis | 0.0000 | 2.301328 | URP |
| Hemopoiesis | 0.0000 | 2.285327 | URP |
| Serine type endopeptidase inhibitor activity | 0.0009 | 2.274775 | URP |
| Molecular transducer activity | 0.0000 | 2.273496 | URP |
| Renal system development | 0.0000 | 2.273188 | URP |
| Glycosaminoglycan binding | 0.0003 | 2.257263 | URP |
| Response to lipid | 0.0000 | 2.257042 | URP |
| Negative regulation of multicellular organismal process | 0.0000 | 2.243461 | URP |
| Cellular response to cadmium ion | 0.0004 | 2.196583 | URP |
| Sulfur compound transport | 0.0010 | 2.192836 | URP |
| Cell adhesion | 0.0000 | 2.185742 | URP |
| Regulation of dna binding transcription factor activity | 0.0000 | 2.180412 | URP |
| Extracellular matrix structural constituent | 0.0010 | 2.17909 | URP |
| External encapsulating structure organization | 0.0000 | 2.173725 | URP |
| Cell motility | 0.0000 | 2.165372 | URP |
| Positive regulation of gene expression | 0.0000 | 2.163244 | URP |
| Peptide cross linking | 0.0003 | 2.14931 | URP |
| Dna binding transcription activator activity | 0.0001 | 2.149197 | URP |
| Modified amino acid transmembrane transporter activity | 0.0030 | 2.142356 | URP |
| Regulation of cell differentiation | 0.0000 | 2.134877 | URP |
| Inorganic anion transmembrane transporter activity | 0.0031 | 2.132865 | URP |
| Growth factor activity | 0.0032 | 2.131501 | URP |
| Negative regulation of developmental process | 0.0000 | 2.107405 | URP |
| Enzyme linked receptor protein signaling pathway | 0.0000 | 2.092945 | URP |
| Toll like receptor binding | 0.0015 | 2.083405 | URP |
| Cargo receptor activity | 0.0101 | 2.074957 | URP |
| Antigen binding | 0.0104 | 2.056613 | URP |
| Response to endogenous stimulus | 0.0000 | 2.048986 | URP |
| Growth factor binding | 0.0047 | 2.04847 | URP |
| Multicellular organismal level homeostasis | 0.0000 | 2.045383 | URP |
| Positive regulation of transcription by rna polymerase ii | 0.0000 | 2.03794 | URP |
| Pattern recognition receptor activity | 0.0092 | 2.010931 | URP |
| Chronic inflammatory response | 0.0122 | 2.000636 | URP |
| Protein trimerization | 0.0120 | 1.964647 | URP |
| Rage receptor binding | 0.0015 | 1.955824 | URP |
| Inorganic anion transport | 0.0109 | 1.916146 | URP |
| Negative regulation of molecular function | 0.0001 | 1.89111 | URP |
| Protein containing complex binding | 0.0001 | 1.851914 | URP |
| Carbohydrate binding | 0.0215 | 1.850225 | URP |
| Growth | 0.0003 | 1.839816 | URP |
| Lipid localization | 0.0030 | 1.792218 | URP |
| Regulation of catalytic activity | 0.0000 | 1.731526 | URP |
| Ribonucleoprotein complex biogenesis | 0.0189 | -1.71469 | DRP |
| Detection of stimulus involved in sensory perception | 0.0226 | -1.88948 | DRP |
| Synaptic signaling | 0.0001 | -1.97663 | DRP |
| Calcium ion import across plasma membrane | 0.0187 | -2.0129 | DRP |

URP – upregulated pathway; DRP – downregulated pathway

**Suplemmentary table 4**. Novel biological pathways identified as consequence of the novel DEGs identified using age-matched samples

| Pathway | FDR | NES | URP/DRP |
| --- | --- | --- | --- |
| Regulation of immune system process | 0.0000 | 1.9646 | URP |
| Cell activation | 0.0000 | 1.8670 | URG |
| Defense response | 0.0000 | 1.8118 | URG |
| Immune response | 0.0000 | 1.7766 | URG |

URP – upregulated pathway; DRP – downregulated pathway

## Supplementary Figures

**
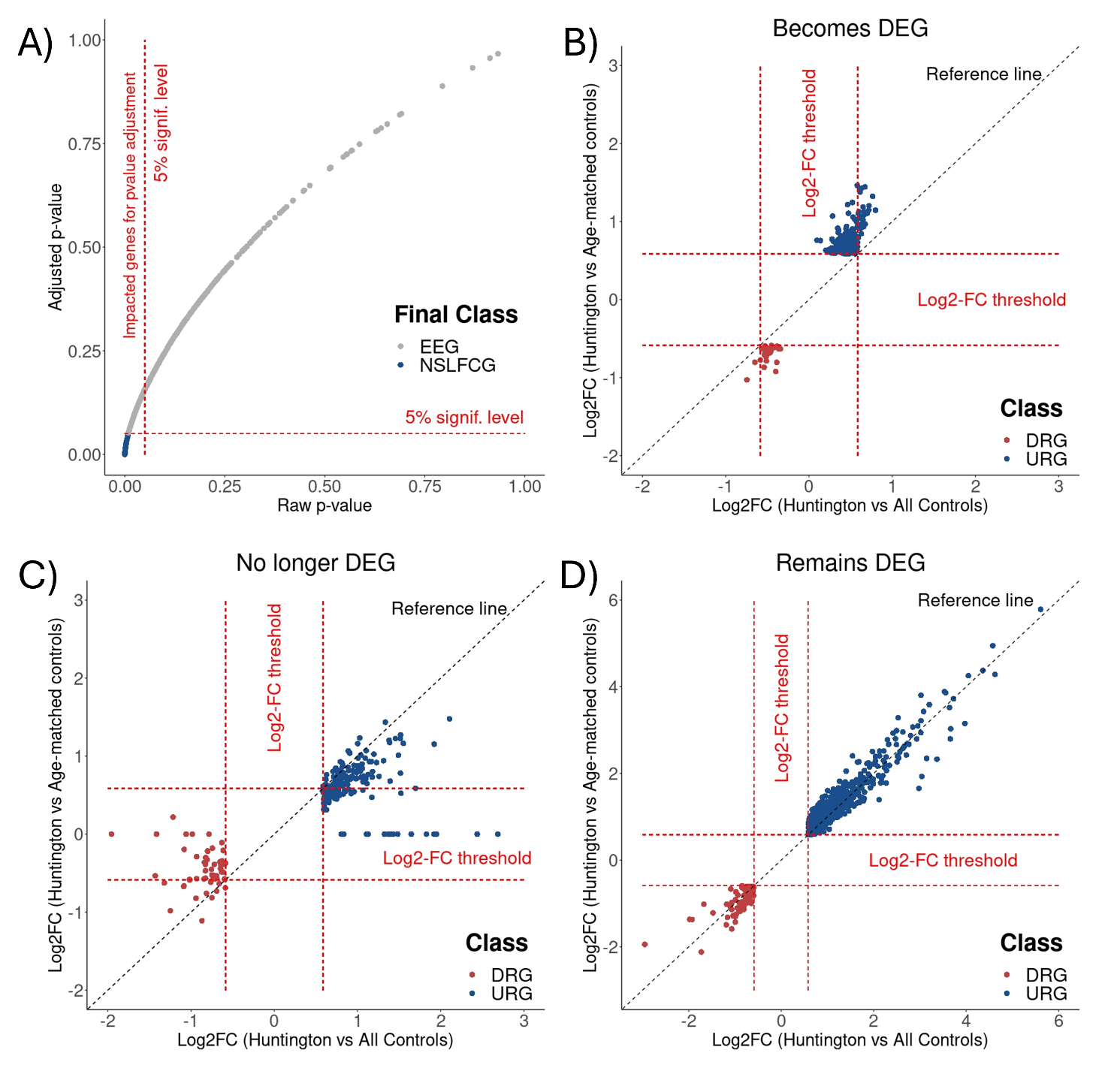
**

**Supplementary Figure 1.** Impact of Age Matching on Differential Expression Analysis: A) The results demonstrate that age matching significantly influences p-value adjustment, leading to the reclassification of a subset of DEGs as NDEGs. B) Changes in Log2FC observed in genes that transitioned to become DEGs with age matching. C) Alterations in Log2FC for genes that were no longer classified as DEGs with age matching. D) Log2FC variations in genes that retained their classification as DEGs despite age matching. This analysis highlights the pivotal role of age matching in not only affecting statistical significance (p-value adjustment) but also in modifying the expression levels (Log2FC) of genes, emphasizing its importance in accurate differential expression assessments.

**Supplemenatry Excel 1**. Demographid data from cases and controls. Data obtained from Labadorf et al. (2015)

**Supplemenatry Excel 2**. Results of functional enrichment analysis (by GSEA) showing the list of DEGs and the biological process (BP) or molecular function (MF) in which they are involved.

**Supplemenatry Excel 3**. Complete list of putative DEGs identified in HD in relation to age-matched controls
